# Secreted frizzled related-protein 2 (Sfrp2) deficiency decreases adult skeletal stem cell function in mice

**DOI:** 10.1038/s41413-021-00169-7

**Published:** 2021-12-02

**Authors:** Luis Fernandez de Castro, Brian J. Sworder, Byron Mui, Kathryn Futrega, Agnes Berendsen, Matthew D. Phillips, Nathan J. Burbach, Natasha Cherman, Sergei Kuznetsov, Yankel Gabet, Kenn Holmbeck, Pamela G. Robey

**Affiliations:** 1 Skeletal Biology Section, National Institute of Dental and Craniofacial Research, National Institutes of Health, Department of Health and Human Services, National Institutes of Health, Bethesda, MD USA; 2 Department of Molecular Medicine, Boston University, Boston, MA USA; 3 School of Dentistry, University of Minnesota—Twin Cities, Minneapolis, MN USA; 4 Department of Anatomy and Anthropology, Sackler Faculty of Medicine, Tel Aviv University, Tel Aviv-Yafo, Israel

**Keywords:** Bone, Bone quality and biomechanics

## Abstract

In a previous transcriptomic study of human bone marrow stromal cells (BMSCs, also known as bone marrow-derived “mesenchymal stem cells”), *SFRP2* was highly over-represented in a subset of multipotent BMSCs (skeletal stem cells, SSCs), which recreate a bone/marrow organ in an in vivo ectopic bone formation assay. SFRPs modulate WNT signaling, which is essential to maintain skeletal homeostasis, but the specific role of SFRP2 in BMSCs/SSCs is unclear. Here, we evaluated *Sfrp2* deficiency on BMSC/SSC function in models of skeletal organogenesis and regeneration. The skeleton of *Sfrp2*-deficient (KO) mice is overtly normal; but their BMSCs/SSCs exhibit reduced colony-forming efficiency, reflecting low SSC self-renewal/abundancy. *Sfrp2* KO BMSCs/SSCs formed less trabecular bone than those from *WT* littermates in the ectopic bone formation assay. Moreover, regeneration of a cortical drilled hole defect was dramatically impaired in *Sfrp2* KO mice. *Sfrp2*-deficient BMSCs/SSCs exhibited poor in vitro osteogenic differentiation as measured by *Runx2* and *Osterix* expression and calcium accumulation. Interestingly, activation of the Wnt co-receptor, Lrp6, and expression of Wnt target genes, *Axin2*, *C-myc* and *Cyclin D1*, were reduced in *Sfrp2*-deficient BMSCs/SSCs. Addition of recombinant Sfrp2 restored most of these activities, suggesting that Sfrp2 acts as a Wnt agonist. We demonstrate that *Sfrp2* plays a role in self-renewal of SSCs and in the recruitment and differentiation of adult SSCs during bone healing. SFRP2 is also a useful marker of BMSC/SSC multipotency, and a factor to potentially improve the quality of ex vivo expanded BMSC/SSC products.

## Introduction

Bone marrow stromal cells (BMSCs, also referred to as bone marrow-derived “mesenchymal stem cells”), are a population of non-hematopoietic, rapidly adherent fibroblastic cells first described by Friedenstein and Owen [reviewed in refs. ^1,2^] Non-clonal strains of BMSCs generate cartilage in an in vitro cell pellet assay, as well as osteogenic cells, hematopoiesis-supportive stroma and marrow adipocytes upon in vivo transplantation with appropriate scaffolds, based on the presence of a subset of skeletal stem cells (SSCs) within bone marrow stromal cells (bmSSCs).^3^ These cells are anatomically, phenotypically and functionally distinct from SSCs within the developing growth plate (gpSSCs), and in the periosteum (pSSCs)^4–8^ (reviewed in ref. ^9,10^).

When a single-cell suspension of freshly isolated human bone marrow is plated at low density, a number of individual BMSCs/SSCs, known as colony-forming unit-fibroblasts (CFU-Fs), rapidly adhere and proliferate, generating colonies. When single colony-derived strains (SCDSs, initiated by a single CFU-F) were transplanted in vivo to form ectopic ossicles, 12.5% of them were able to generate a complete bone-marrow organ and were designated multipotent SCDSs (M-SCDSs) containing bmSSCs. The remaining strains either formed bone tissue but did not support hematopoiesis (66.7%, B-SCDSs), or formed fibrous tissue (20.8%, F-SCDSs). The transcriptomic profile of aliquots of the M-SCDSs and F-SCDSs cultures used for in vivo transplantation were compared, and *SFRP2* gene was highly and consistently over-represented in M-SCDSs, suggesting its potential role in SSCs.^11^

SFRP2 is one of five members of the Secreted Frizzled-Related Protein (SFRP) family [FRZB, SFRP1, 2, 4, 5 (also known as COMP)]. SFRPs share two conserved domains separated by a short linker region: a N-terminal Cysteine Rich Domain (CRD), with homology to the WNT-binding site in Frizzled, and a C-terminal Netrin-related Domain (NTR) with homology to Laminin.^12^ Members of the SFRP family are reported to be modulators of the Wnt signaling pathway, which has emerged as a critical component in bone modeling, remodeling and regeneration (reviewed in ref. ^13–15^) Reduction in WNT signaling can result in loss of bone mass,^16,17^ while excessive activation has the opposite effects.^18–20^ Indeed, WNT pathway activation improves healing of transcortical defects through SSC differentiation into osteoblasts.^21,22^ Fine-tuning of this pathway is controlled by combinations of Frizzled receptors and co-receptors; as well as factors that bind to WNT and/or its receptors, including SFRPs, and an ever-increasing list of ligands.

Previous studies have reported negative effects of SFRP2 on WNT signaling in osteoblast differentiation and bone formation [e.g.,^23,24^] However, many of these studies solely utilized cell line models. One study of *Sfrp2-*deficient (KO) mice showed subtle differences in long bone length in the distal portion of the appendicular skeleton, leading to mild brachydactyly, the cause of which was determined to be decreased chondrocyte proliferation and delayed maturation.^25^ However, a comprehensive skeletal analysis was not performed to determine other potential changes in the skeleton of *Sfrp2* KO mice.

Although previously regarded as inhibitors of WNT signaling, the action of SFRPs is now recognized to be more complex. SFRPs may operate as either WNT inhibitors or activators depending on the cell type, receptor expression pattern, and the local presence of other WNT-related or unrelated factors.^26–28^ Motivated by the high *SFRP2* expression observed in human multipotent BMSCs/SSCs, we obtained *Sfrp2* KO mice to investigate the role of this gene in postnatal BMSCs/SSCs during bone regeneration, and its modulation of the Wnt pathway during their differentiation.

## Results

### *Sfrp2* expression is enriched in *Lepr*^*+*^ bone marrow-derived stromal stem/progenitor cells in mice

**Fig. 1:**
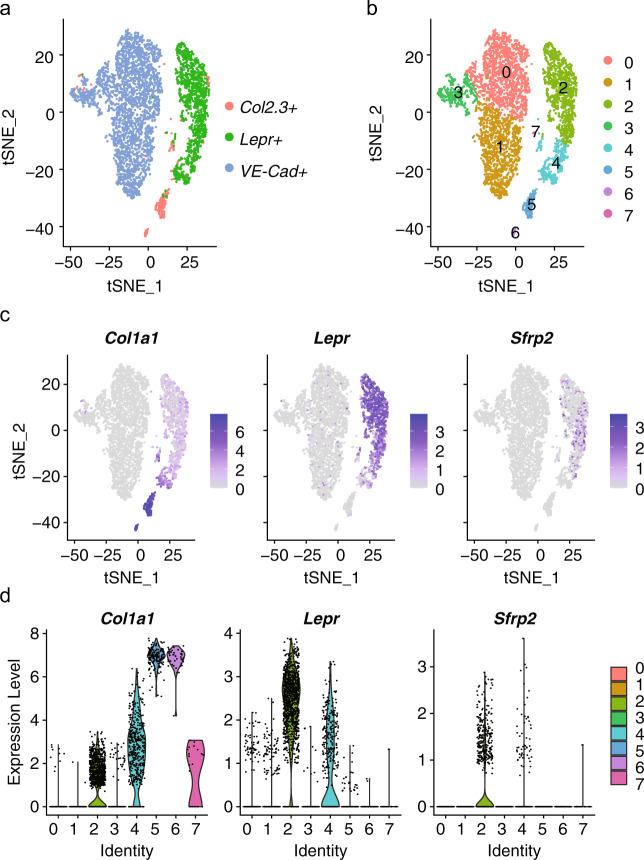
Analysis of scRNA-seq data from previously published datasets^29^ that assessed non-hematopoietic cell lineages of mouse bone marrow in steady state conditions. **a** tSNE plot of cre-induced osteoblasts (*Col2.3*^*+*^; red), mesenchymal stromal cells (*Lepr*^+^; green), and vascular cells (*VE-Cad*+; blue) and (**b**) associated clusters. **c**, **d** *Sfrp2*-expressing cells were found to be significantly enriched in cluster 2, a cluster containing the *Lepr*^+^ skeletal stem/progenitors (termed “mesenchymal stromal” cell population by the authors) that also exhibited the greatest number of cells with a high magnitude of *Lepr* expression

We analyzed a publicly available single-cell RNA-seq datasets^29^ to determine the expression of *Sfrp2* in the mouse non-hematopoietic bone marrow compartment. These datasets were generated using cells that were isolated from lineage-specific Cre-transgenic mice crossed with a knock-in reporter strain, Rosa26 locus (LoxP-tdTomato). The datasets included cells marked by the expression of *VE-Cad*, *Lepr* and the *Col 2.3* *kb* promoter (*Col1a1*) to identify vascular, bone marrow-derived stem/progenitor cells (termed “mesenchymal stromal cells” by the authors), and osteoblasts at steady state, respectively (see^29^ for experimental details). We performed our own independent analysis of the data using the Seurat package in R. We found that *Sfrp2*^+^ cells were localized within the *Lepr*^+^ population, specifically in cluster 2 (average log2FC of 0.89 and *P*-adjusted value of 8.7 × 10^−163^) (Fig. 1). These results indicate that, as in human bone marrow, *Sfrp2* is expressed by a subset of bone marrow-derived skeletal stem/progenitor cells.

### *Sfrp2* KO mice exhibit normal bone structure and morphometric features

**Fig. 2:**
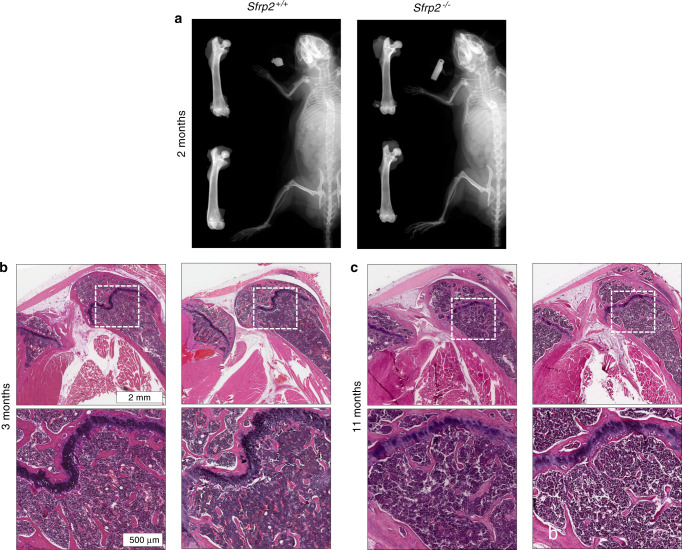
*Sfrp2* KO mice do not exhibit radiological or histological skeletal differences compared with *WT* mice. **a** Representative X-ray images of 2-month-old male mice femora and full skeletons. **b**, **c** Micrographs (top row) of mid-sagittal H&E-stained sections of the knee joint, with femora on the right and tibiae on the left, and high magnification micrographs of the sub-metaphyseal area of femora (bottom row), of male mice at 3 months (**b**), and 11 months (**c**)

The skeletal phenotype of *Sfrp2* KO mice was first characterized using a variety of imaging modalities. In addition to the mild brachy-syndactyly already described in this model (not shown),^25^ high-definition X-rays of these mice did not show any additional skeletal phenotype at 2 months of age (Fig. 2a). Bone mineral density measured by X-ray absorptiometry (DEXA) was not altered (data not shown). Femoral analyses by µCT and histomorphometry at 3 and 11 months were also unremarkable and did not discriminate *WT* from *Sfrp2* KO mice (Supplementary Fig. 1). Cranial anatomy of 3-month-old animals analyzed by µCT was likewise unremarkable (Supplementary Fig. 2A). Calvariae were stained for TRAP activity as an approximation of osteoclastic activity. The cranial sutures [a common area of abnormal bone resorption in mice^30^] were not affected by the absence of *Sfrp2* (Supplementary Fig. 2B). To better examine changes in soft tissues in relationship to mineralized tissues, *WT* and *Sfrp2* KO femora were examined histologically. Again, there was no histological evidence of changes in *Sfrp2* KO femora compared with *WT* femora at 3 months and 11 months of age (Fig. 2b, c).

### Sfrp2-deficient BMSCs/SSCs have reduced colony-forming efficiency (CFE)

**Fig. 3:**
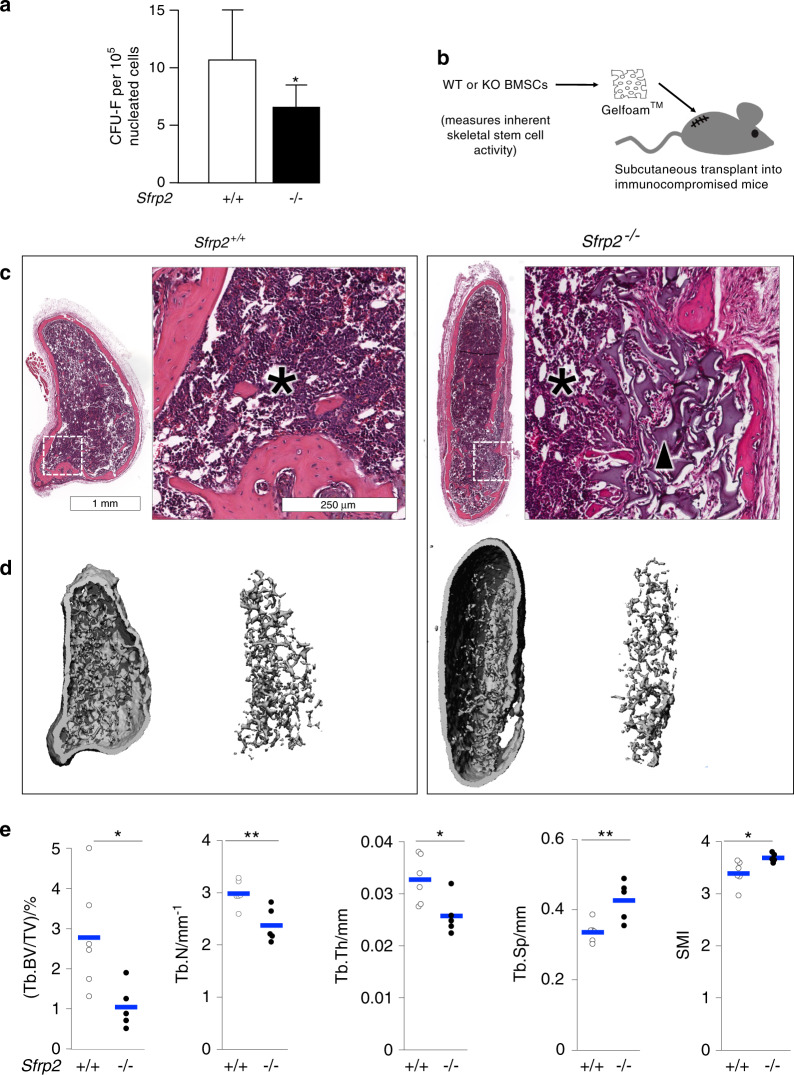
*Sfrp2*-deficient BMSCs/SSCs have lower colony-forming efficiency and reduced osteogenic differentiation in ectopic bone transplants. **a** Colony-forming efficiency assay showing colony-forming units-fibroblasts (CFU-F) per 100 000 nucleated bone marrow cells from *WT* and *Sfrp2* KO littermates. **b** Gelatin sponges were loaded with 2 × 10^6^ BMSCs/SSCs depleted of hematopoietic cells and transplanted into subcutaneous pockets in immunocompromised mice for 8 weeks. **c** H&E-stained sections of the transplants. *WT* BMSCs/SSCs formed a complete bone/marrow organ, with cortical and trabecular bone, stroma that supports hematopoiesis of mouse origin (black asterisk). *Sfrp2* KO BMSCs/SSCs were less osteogenic; transplants showed residual gelatin sponge scaffold material (black arrowheads, far right panel). **d** µCT reconstructions of transplants and their trabecular compartments. **e** µCT analysis of trabecular bone. Compared with *WT* transplants, *Sfrp2* KO transplants had fewer, thinner trabeculae that were more separated and had abnormal geometry (SMI). (Tb.BV/TV)/% Trabecular bone volume/Total volume (%), Tb.Th Trabecular Thickness, Tb.Sp Trabecular Separation, Tb.N Trabecular Number, SMI Structure Model Index. Individual values are shown as five white (*WT*) or six black circles (*Sfrp2* KO), and blue bars represent the mean. *P < 0.05; **P < 0.01

An initial characterization of the cellular phenotype of the *Sfrp2* KO mice was the determination of colony-forming efficiency (CFE) of single-cell suspensions of bone marrow, a measure of the number of colony-forming units-fibroblasts (CFU-Fs). The number of CFU-Fs in bone marrow is currently the closest approximation of the number of bmSSCs present in the BMSC population.^31^ When cultured at clonal density, bone marrow cells from *Sfrp2* KO mice showed a pronounced decrease (~40%) in the number of colonies derived from single CFU-Fs after 14 days of culture (Fig. 3a).

### *Sfrp2* KO BMSCs have a decreased ability to form a bone/marrow organ de novo in vivo

A well-established in vivo assay that measures the intrinsic ability of bmSSCs within BMSC populations to form a bone/marrow organ is by transplantation of the cells along with an appropriate scaffold into a subcutaneous pocket in immunocompromised mice (in vivo ectopic ossicle).^32^ Establishment of a complete bone/marrow organ by transplanted cells is completely dependent on the presence of the bmSSC within the transplanted BMSC population^2^ (Fig. 3b). For this purpose, *WT* and *Sfrp2* KO BMSCs/SSCs were established in culture. No differences in growth rate were observed between the two genotypes (data not shown). At passage 2 or 3, the cells were depleted of CD45^+^/CD11b^+^ hematopoietic cells before being absorbed into Gelfoam^TM^ (gelatin) sponges and transplanted into subcutaneous pockets in immunocompromised mice. Eight weeks later, the ectopic ossicles were retrieved, fixed, scanned by µCT for morphometric analysis, and examined histologically by H&E staining. Transplanted *WT* cells developed a typical ectopic bone/marrow organ (Fig. 3c, left) composed of donor-derived bone, marrow stroma and host-derived hematopoietic cells (asterisk, Fig. 3c, left). The same number of transplanted *Sfrp2* KO BMSCs failed to fully resorb the gelatin scaffold material (arrowhead in Fig. 3c, right), and did not completely establish a normal ossicle. µCT analysis (Fig. 3d, e) showed less trabecular bone in *Sfrp2* KO transplants (Tb.BV/TV), with fewer, thinner and more widely spaced trabeculae (Tb.N, Tb.Th, and Tb.Sp, respectively), that was also structurally altered (structure model index, SMI) (Fig. 3e).

### *Sfrp2* KO mice display reduced intramembranous bone regeneration in response to injury

**Fig. 4:**
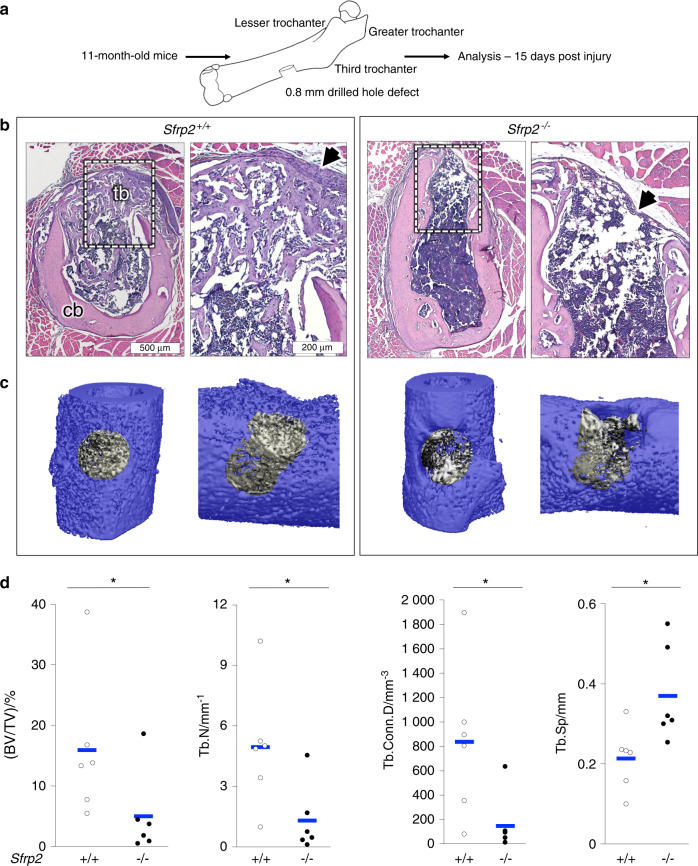
*Sfrp2* KO mice exhibit a bone regeneration defect in vivo. **a** Drilled bone defect assay. **b** Mid-transverse sections of the bone defect stained with H&E. Periosteal regeneration was evident in *WT* mice, while *Sfrp2* KO mice exhibited little periosteal response (black arrows). Tb trabecular bone, Cb cortical bone. **c** µCT reconstructions showing in gray the drilled volume evaluated. **d** Compared with *WT*, the defects in *Sfrp2* KO mice had less bone, fewer trabeculae with less connectivity that were more widely spaced. (Tb.BV/TV)/% Trabecular bone volume/Total volume (%), Tb.N Trabecular number, Tb.Conn.D Trabecular connectivity density, Tb.Sp Trabecular separation. Individual values are shown as six white (*WT)* or five black dots (*Sfrp2* KO), and blue bars represent the mean. *P < 0.05 vs. *WT*

Based on the finding of decreased bone formation by the *Sfrp2* KO BMSCs in the in vivo ectopic ossicle assay, we sought to determine if a similar intrinsic defect could be observed in a minor in vivo injury. For this purpose, a drilled hole defect was created in the left femur of *Sfrp2* KO and *WT* littermate mice to assess their intramembranous cortical bone regeneration (Fig. 4a, method modified from Monfoulet et al.^33^) keeping in mind that there was no periosteum overlying the defect (therefore, no cartilaginous callus is formed). Fifteen days after surgery, the cortical bone defect and underlying marrow was filled with trabecular bone in *WT* mice (Fig. 4b, left), but next to no new bone was visible in *Sfrp2* KO mice defects (Fig. 4b, right). In addition, a regenerated periosteum was greatly thickened in the *WT* defect, but not in the *Sfrp2* KO defect (Fig. 4b, large black arrows). Indeed, by µCT analysis of the regeneration site (Fig. 4c), we observed a three- to five-fold reduction in bone volume (BV/TV), in trabecular number (Tb.N) and trabecular connectivity density (Tb.Conn.D) in *Sfrp2* KO compared with their WT littermates, while the trabecular spacing (Tb.Sp) was almost twice as high (Fig. 4d).

### Sfrp2 deficiency reduces BMSC/SSC osteogenic differentiation in vitro

**Fig. 5:**
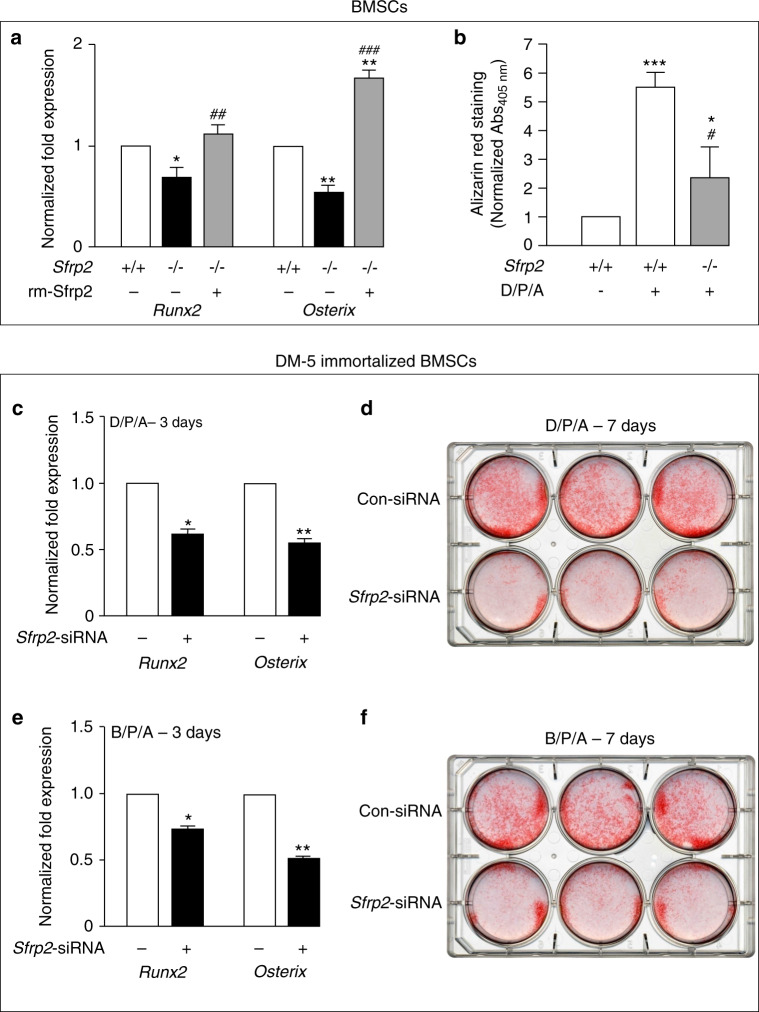
Sfrp2 deficiency leads to a reduced osteogenic differentiation capacity of BMSCs/SSCs in vitro. **a** After 3 days of osteogenic differentiation with dexamethasone, β-glycerol phosphate and L-ascorbic acid 2-phosphate (D/P/A), *Sfrp2* KO BMSCs/SSCs showed less expression of *Runx2* and *Osterix* (vs. *Gapdh*) than *WT* BMSCs/SSCs. Addition of exogenous rm-Sfrp2 (1 μg·mL^−1^) normalized *Runx2* expression, and increased *Osterix* expression above *WT* levels. **b** After 21 days of osteogenic differentiation (D/P/A), *Sfrp2* KO BMSC/SSC cultures were less mineralized than *WT* cultures, as measured by alizarin red S staining and absorptiometry at 405 nm. *P < 0.05; **P < 0.01; ***P < 0.01 vs. *WT* BMSCs/SSCs; ^#^*P* < 0.05; ^##^*P* < 0.01; ^###^*P* < 0.01 vs. *Sfrp2* KO BMSCs/SSCs^.^ **c**–**f** DM-5, an immortalized BMSC/SSC line that retains the ability to form an ectopic ossicle upon transplantation, was transfected with scrambled (Con-siRNA) or *Sfrp2*-siRNA. After 3 days of osteogenic differentiation with either D/P/A in (**c**), or BMP-2, β-glycerol phosphate and L-ascorbic acid 2-phosphate (B/P/A) in (**e**), cells silenced for *Sfrp2* expression showed decreased expression of *Runx2* and *Osterix* (vs. *Gapdh*). After 7 days of osteogenic differentiation with D/P/A or B/P/A, cells silenced for *Sfrp2* expression showed decreased calcium accumulation as shown in alizarin red S stains in (**d**, **f**). Data represent mean ± SD. *P < 0.05; **P < 0.01 vs. Con-siRNA-treated cells

In order to determine the reason for the lack of bone formation in both in vivo models, we next assessed *Sfrp2* KO and *WT* BMSCs for their osteogenic capacity in vitro. At Passage 3 or less, cultures were depleted of hematopoietic CD45^+^ and CD11b^+^ cells and induced towards osteogenesis using medium containing dexamethasone/β-glycerophosphate/ascorbic acid/ (D/P/A). Three days after induction, *Sfrp2* KO cultures showed a significantly decreased expression of the early osteogenic transcription factors, *Runx2* and *Osterix* (Fig. 5a). Addition of recombinant murine Sfrp2 (rm-Sfrp2) restored the expression levels of *Runx2* and *Osterix* (Fig. 5a). Three weeks after induction, *Sfrp2* KO cultures showed a lower level of calcium accumulation as measured by alizarin red staining, dissolution and spectroscopic measurement (Fig. 5b).

These observations were replicated with an independent assay using an immortalized murine BMSC cell line, DM-5, that maintains the ability to form a complete ectopic ossicle upon in vivo transplantation.^34,35^ Like *Sfrp2* KO cells, DM-5 cells silenced for *Sfrp2* expression (Fig. 5c–f, Supplementary Fig. 3), showed a significant reduction in the expression of *Runx2* and *Osterix* 3 days after initiation of osteogenic differentiation in vitro using either 10 nmol·L^−^^1^ dexamethasone (Fig. 5c), or 100 ng·μL^−1^ BMP-2 (Fig. 5e), in addition to 100 μmol·L^−1^ ascorbic acid 2-phosphate and 1 mmol·L^−1^ β-glycerol phosphate. Silenced cells in both conditions showed a decreased calcium accumulation at 7 days as well (Fig. 5d, f).

### Sfrp2 deficiency reduces Wnt signaling in BMSCs/SSCs

**Fig. 6:**
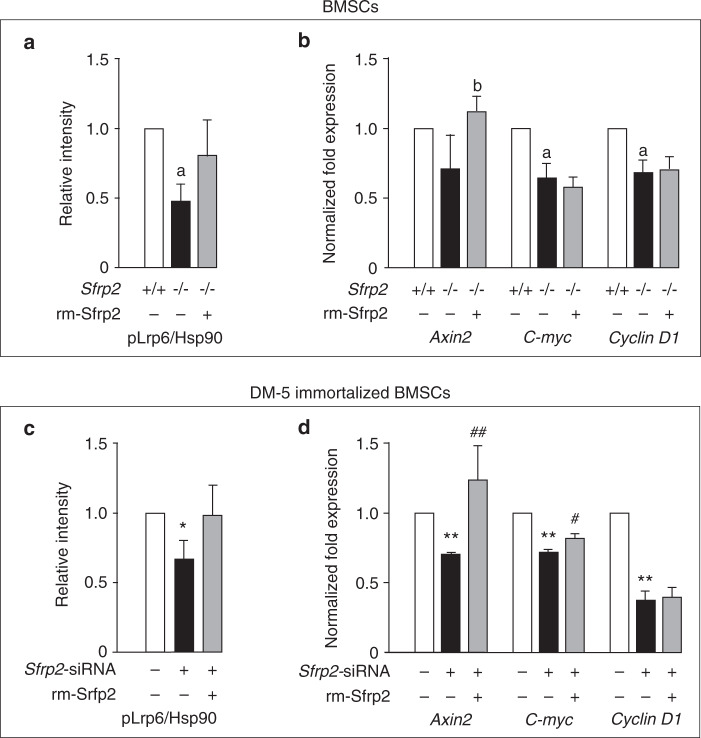
*Sfrp2* deficiency reduces Wnt signaling in murine BMSC/SSCs. **a**, **b** BMSCs/SSCs from WT and *Sfrp2* KO mice were cultured, depleted of hematopoietic cells and treated with 1 μg·mL^−1^ rm-Sfrp2 or vehicle. *C,D* DM5 cells were transfected with scrambled (Con-siRNA) or *Sfrp2*-siRNA and treated with 1 μg·mL^−1^ rm-Sfrp2 or vehicle. **a**, **c** Lrp6-Ser1490 phosphorylation, as measured by western blot densitometry versus HSP90, was decreased in Sfrp2-deficient cultures and trended to normalize 5 h after the addition of rm-Sfrp2. It is possible that the reported levels of pLrp6 (Ser1490) also include pLrp5 (ser1493), as per the manufacturer’s description. The antibody used is expected to also detect this highly similar epitope. **b**, **d** Expression of Wnt target genes *Axin2*, *C-myc* and *Cyclin D1* (vs. *Gapdh*) was decreased in *Sfrp2*-deficient cultures and partially normalized 24 h after the addition of rm-Sfrp2. Data are presented as the mean of three or more independent experiments ± SD. ^a^*P* < 0.05 vs. *WT* BMSCs/SSCs, ^b^*P* < 0.05 vs. *Sfrp2* KO BMSCs/SSCs. *P < 0.05; **P < 0.01 vs. *Con*-*siRNA* treated cells; ^#^*P* < 0.05, ^##^*P* < 0.01 vs. *Sfrp2-siRNA* treated cells

Wnt signaling is known to positively regulate osteogenesis, and it has been previously suggested that Sfrp2 is a Wnt antagonist. On the contrary, our data suggested that Sfrp2 is required for normal osteogenesis, both in vivo and in vitro. Consequently, we examined the Wnt signaling pathway in *WT* and *Sfrp2* KO BMSCs/SSCs depleted of hematopoietic cells to determine if Sfrp2 is a positive regulator of immature skeletal stem and progenitor cells. We demonstrated that, rather than increased, phosphorylation of the Wnt co-receptor, Lrp6, was reduced in *Sfrp2* KO cells (Fig. 6a). It is possible that the reported levels of pLrp6 (Ser1490) also include pLrp5 (ser1493), as per the manufacturer’s description. The antibody used is expected to also detect this highly similar epitope. Furthermore, the expression of Wnt downstream target genes *Axin2, C-myc*, and *Cyclin D1*, were also reduced in *Sfrp2* KO cells, although the reduction in *Axin2* was not statistically significant (Fig. 6b). Addition of rm-Sfrp2 showed a non-significant trend toward a rescue in the level of pLrp6 after 5 h of treatment (Fig. 6a), and increased *Axin2* after 24 h, but did not restore *C-myc* or *Cyclin D1* (Fig. 6b).

Similar results were obtained when we replicated the assays in an independent system using immortalized DM-5 BMSCs/SSCs that were treated with *Sfrp2*-siRNA. pLrp6 was reduced in silenced cells (Fig. 6c), as was *Axin2, C-myc*, and *Cyclin D1* (Fig. 6d). Treatment of the silenced cells with rm-Sfrp2 for 5 h showed a trend to increase pLrp6, and at 24 h, restored *Axin2* expression, with a very modest increase in *C-myc*, but no increase in *Cyclin D1* expression (Fig. 6c, d).

## Discussion

Adult BMSCs and their bmSSC subset have a high therapeutic potential for regenerating new bone, reflected by an abundance of preclinical data. However, many of their theoretical applications in tissue engineering and reconstructive surgery, whereby the cells themselves regenerate lost skeletal elements, remain problematic. This is due, in part, to the difficulty in manufacturing cell products with sufficient numbers of cells with consistent biological activity required for these applications. Extensive ex vivo expansion of BMSCs/SSCs decreases their differentiation capacity in vivo, as the relative abundance of SSCs within BMSC populations progressively decrease with increasing passage number,^36^ (Kuznetsov and Robey, unpublished results). More recently, we showed that even in clinical-grade products, BMSC/SSC differentiation capacity varied greatly depending on the manufacturing method utilized.^37^ In order to develop better in vitro expansion methods able to produce a high yield of multipotent cultured BMSCs/SSCs, it is essential to gain insight into the regulation of SSC self-renewal and to identify biological markers that predict the differentiation potential of cultured BMSCs/SSCs. In a previous study using in vivo ectopic bone formation assays, we demonstrated that: (1) only a small proportion (12.5%) of bone marrow CFU-F-derived cell strains are truly multipotent (M-SCDSs), and can differentiate not only into osteoblasts/osteocytes but also into hematopoiesis-supporting stroma and marrow adipocytes;^11,38^ and (2) these cells consistently exhibited a high expression of *SFRP2*.^11^ This not only supports the use of this WNT modulator as a marker of BMSC multipotency, but also as a potential regulator of bmSSC self-renewal.

Supporting the latter hypothesis, using publicly available datasets of murine bone marrow single-cell RNA sequencing, performed by Tikhonova et al. we observed that *Sfrp2* is co-expressed in *Lepr*^+^ cells, a well-known marker of adult SSCs in mice^29^ (Fig. 1). Moreover, we found that bone marrow from *Sfrp2* KO mice had fewer CFU-Fs than their *WT* littermates (Fig. 3a). However, these mice were only reported to have a minor cartilage defect during fetal development, leading to a subtle brachydactyly phenotype.^25^ Since this study did not include a detailed phenotyping of the adult skeleton of *Sfrp2* KO mice, we thoroughly studied their skeleton using X-Rays, DEXA (not shown), µCT and histology. Our analyses failed to reveal differences between either young (2–3-month-old) or old (11-month-old) sex-matched *Sfrp2* KO and *WT* littermates (Fig. 2, Supplementary Figs. 1, 2), demonstrating that Sfrp2 is not essential for fetal or postnatal modeling and remodeling of the skeleton in mice. Interestingly, *Sfrp1* KO mice are also born normal, but *Sfrp1* and *Sfrp2* double deficiency causes lethality, shortening of the thoracic region and abnormal limb morphogenesis.^39^ This indicates a high degree of redundancy in the function of Sfrp1 and 2 during fetal development. Of note, we observed a strong up-regulation of *Sfrp1* and to a lesser degree, of *Sfrp4*, in cultured *Sfrp2*-KO BMSCs (Supplementary Fig. 5).

SFRPs were initially described as negative WNT modulators.^40^ However, their role in WNT signaling appears to be complex and incompletely understood. SFRPs have been shown to act as antagonists by sequestering WNT via their NTRs and/or CRDs [reviewed in,^41,42^] or by direct binding to Frizzled receptors through their CRDs,^43^ However, SFRPs are also suggested to be agonizts by stabilizing extracellular WNT and WNT-Frizzled complexes,^44^ or by direct activation of Frizzled,^45^ or enhancement of ligand-induced signaling.^46^ Different SFRPs may bind to each other, leaving WNT free to promote signaling [proposed in.^42^] More recently, SFRPs have been reported to act intracellularly by directly binding to β-catenin to repress or activate TCF4 recruitment.^12^ Lastly, WNT-independent actions such as regulation of BMP signaling pathways have also been suggested.^47,48^ In light of this complexity, it is not unexpected that the literature reflects diversity in the downstream effects of SFRPs. A current interpretation is that SFRPs’ mechanisms of action are highly dynamic, and vary greatly depending on the cell type, differentiation stage and microenvironment.^26^

In our in vitro experiments, *Sfrp2* deficiency was associated with a reduced osteogenic differentiation by either dexamethasone/ascorbate/β-glycerophosphate or BMP-2 osteogenic medium (Fig. 4). Adding rm-Sfrp2 to *Sfrp2* KO BMSC/SSC cultures restored the expression of osteogenic markers. This contrasts with previous data showing decreased BMP2-mediated osteogenic differentiation of MC3T3 cells after the addition of exogenous Sfrp2.^49^ However, Boland et al. showed that *SFRP2* is upregulated during osteogenic differentiation of human BMSCs,^50^ supporting the notion of a complex, differentiation stage-dependent action of SFRP2.

In order to determine if the decrease in osteogenic differentiation in the face of *Sfrp2* deficiency was due to a decrease in Wnt signaling, we used two different experimental approaches: (1) BMSCs/SSCs derived from *Sfrp2* KO and *WT* littermates, and (2) knocking down *Sfrp2* mRNA expression in immortalized DM-5 cells in vitro (Supplementary Fig. 4). In both cases, abrogation of *Sfrp2* expression in undifferentiated osteoprogenitors caused a decrease in phosphorylation of the Wnt co-receptor, Lrp6, and downregulation of Wnt target genes *Axin2, C-myc*, and *Cyclin D1* in vitro, suggesting that Sfrp2 activates Wnt canonical pathway. Addition of rm-Sfrp2 partially restored pLrp6 and completely restored *Axin2*, but did not restore *C-myc* or *Cyclin D1*, possibly due to the 24 h timepoint used to assess the effects of rm-Sfrp2 restoration on gene expression (Fig. 6).

Our observations of decreased osteogenesis and Wnt activation in *Sfrp2*-deficient BMSCs/SSCs in vitro can be the result of an overall activating role of *Sfrp2* in Wnt modulation in our models, but it can also reflect that the increased levels of Sfrp1 and Sfrp4 observed in *Sfrp2* KO BMSCs/SSCs (3-fold and 1.2-fold respectively, Supplementary Fig. 4) caused increased inhibition of Wnt pathway and osteogenesis. Yet, these two explanations are not mutually exclusive. Both Sfrp1 and Sfrp2 have been reported as Wnt inhibitors.^51,52^ These data suggest that while Sfrp1 and Sfrp2 have many overlapping effects, Sfrp2 has activities that are unique with regard to Wnt signaling. In any event, these possibilities are in agreement with the increasing notion that SFRPs and other WNT modulators are a part of a highly dynamic system that behaves differently depending on the model system used, and is very much influenced by the cellular context and differentiation stage. This highlights the need for in vivo assessment of Sfrp2 in the function of skeletal stem cells and osteoprogenitors. With this aim in mind, we carried out two in vivo assays to study different aspects of the role of Sfrp2 in BMSCs/SSCs.

Producing ectopic bone/marrow organoids with BMSCs/SSCs allows for the specific measurement of BMSC/SSC multipotency. The ability of *Sfrp2* KO and *WT* BMSCs to differentiate into a complete ectopic bone/marrow organ was assessed independently of the systemic effects of *Sfrp2* deficiency, as *Sfrp2* KO and *WT* cells were transplanted into an independent organism (a *WT* host mouse). *Sfrp2*-deficient BMSCs/SSCs showed a dramatic reduction in their ability to generate a bone *de novo*, as evidenced by the presence of un-resorbed gelatin scaffold detected by histology, and abnormal µCT measurements. Interestingly, *Sfrp2* deficiency in BMSCs/SSCs did not seem to significantly affect the support of hematopoietic marrow formation in these transplants, which derives from host hematopoietic cells growing on donor stroma (Fig. 3). Consistent with our in vivo ossicle results, *Sfrp2* overexpression in stem cells from the apical papilla (SCAPs) exhibited a superior tissue regeneration capacity in a periodontitis/regeneration model in miniature pigs,^53^ and subcutaneous transplantation of *Sfrp2* knockdown SCAPs (SCAPs of teeth) demonstrated less dentin-like tissue formation than normal cells.^54^

The drilled mid-diaphyseal femur defect is a relatively minor injury that resolves primarily by intramembranous ossification, with a lower involvement of inflammation, vascularization and resorption than in fracture models driven by endochondral ossification.^33^ Consequently, this assay explores the effects of *Sfrp2* deficiency more specifically on the mobilization and osteogenic differentiation of both bmSSCs and periosteal SSCs (pSSCs). *Sfrp2* KO mice were virtually unable to repair this minor injury. *Sfrp2* deficiency negatively affected the mobilization and differentiation capacity of SSCs. Unlike *WT* mice, *Sfrp2* KO mice exhibited no periosteal thickening (Fig. 4b, black arrows), showing a near-complete absence of periosteal SSC (pSSC) response to the injury. While it is more challenging to directly observe a bmSSC response, there was a complete absence of intramedullary trabeculae in the defect. There was a threefold decrease in de novo bone formation in the drilled area as assessed by µCT analysis (Fig. 4c), along with a very low number of trabeculae present only at the margin of the injury in the *Sfrp* KO mice. In this model, it is possible that global *Sfrp2* deficiency could be affecting SSC-independent processes occurring during bone regeneration, such as vascularization, thereby contributing to the failure to regenerate this minor defect. However, the inability of the *Sfrp2* KO mice to heal this minor defect, along with the inability to create a complete bone/marrow organ from their BMSCs/SSCs in the ectopic ossicle model, can be clearly linked to the decrease in CFE of BMSCs/SSCs. This is suggestive of a decrease in self-renewal of the bmSSCs in these mice, and most likely in the self-renewal of the pSSCs, based on the lack of periosteal regeneration. In addition to a decrease in self-renewal and bone regeneration in vivo, the *Sfrp* KO BMSC/SSC population also displayed decreased osteogenic differentiation in vitro, due to a decrease in WNT signaling.

Characterizing autocrine/paracrine factors controlling SSCs self-renewal has important implications for the field of regenerative medicine. Cultured BMSCs/SSCs can potentially repair large segments of bone that are unlikely to adequately heal due to trauma, surgical resection of cancer or skeletal pathologies, but the cell numbers necessary for this goal requires extensive expansion of these cells in vitro. In order to produce enduring regenerated bone, it is essential to guarantee the presence of self-renewing SSCs within BMSC/SSC cultures.^55^ Based on our results, *Sfrp2* is dispensable for the development and maintenance of the mouse skeleton in an unchallenged scenario, but appears to be essential for the mobilization and differentiation of bmSSCs and pSSCs into functional osteogenic cells during bone repair and regeneration. Measuring the expression levels of *SFRP2* in clinical-grade cultured BMSCs could be useful in predicting their subsequent in vivo proliferation and differentiation in tissue engineering applications. Further studies are needed to unravel the intricacies of the role of *Sfrp2* in adult SSC self-renewal and function in response to injury.

## Materials and methods

### Determining *Sfrp2* expression in previously published scRNA-seq data

To determine the expression of *Sfrp2* cells in the mouse non-hematopoietic bone marrow compartment, we analyzed a publicly available single-cell RNA-seq datasets from the GEO series GSE108892.^29^ These datasets were generated using cells that were isolated from lineage-specific Cre-transgenic mice crossed to a knock-in reporter strain, Rosa26 locus (LoxP-tdTomato). The experimental details for generating these datasets were previously published by Tikhonova et al.^29^ and include cells from the following transgenic mouse crosses: VE-Cad-cre;LoxP-tdTomato (VE-Cad–tdTomato) (GSM2915577) for vascular cells; LEPR-cre;LoxP-tdTomato (LEPR–tdTomato) (GSM2915576) for bone marrow-derived stem/progenitor cells (termed “mesenchymal stromal cells” by the authors); and COL2.3-cre;LoxP- tdTomato (COL2.3–tdTomato; note, COL2.3 refers to the 2.3 kb of the Col1a1 promotor) (GSM2915575) for osteoblasts.

The datasets were imported into Seurat package version 4.0.1 in R^56^ and standard workflows were followed with default settings as described in the Seurat online tutorials (https://satijalab.org/seurat/). We filtered out cells that had less than 200 or more than 6 000 genes, and more than 5% mitochondrial counts using the subset function. The datasets were integrated using the Seurat built-in integration functions. Blood cell lineage clusters were identified based on the expression of Ptprc, Lyz2, or hemoglobin genes, and removed by subsetting. After removal of blood cells, the data was r-eclustered and tSNE plots generated. This process resulted in seven clusters for which differentially expressed genes were calculated.

### *Sfrp2* KO mice

*Sfrp2* KO mice were kindly provided by Drs. Roy Morello and Brendan Lee at the Baylor College of Medicine, Houston, TX,^25^ and genotyped by touchdown PCR and electrophoresis in 2% agar using the following primers (derived from NCBI Reference Sequence NC_000069.7): *Wildtype* (*WT*) forward 5′-GAGGTGAAAGAGGTTGGTCGT-3′, *Sfrp2* KO forward 5′-TTGAGCCCGGTGTTACTGGAG-3′ and common reverse 5′-AAACCTTATGACCTCCTGTGAGG-3′. The *Sfrp2* KO amplicon is approximately 700 nt and the *WT* amplicon is 800 nt. This study was conducted under an institutionally approved protocol in accordance with the NIH/NIDCR Animal Care and Use guidelines (#13-687).

### X-ray and microcomputed tomography (µCT) analyses

To characterize the bone phenotype of *Sfrp2* KO mice, whole 8-week-old mice and dissected femora were imaged using an MX-20 X-ray device (Faxitron, Tucson, AZ). Samples were exposed for 15 s at 30 kV using Kodak X-Omat film, which was then electronically scanned. Skulls, femora and ectopic transplants were imaged by microcomputed tomography (µCT, 70 kVp, 85 µA, 0.3 s integration time, 1 000 µm^3^ voxel resolution, Scanco Medical AG, Brüttisellen, Switzerland). Intact femora (3 months and 11 months, males and females) were analyzed for histomorphometric parameters as previously described.^57,58^

### Tissue processing and histology

Anesthetized mice were euthanized by terminal vascular perfusion and fixation by sequentially injecting 10 mL of ice-cold PBS and 10 mL of ice-cold 4% formaldehyde into the left ventricle of the heart [modified from ref. ^59^]. Samples were then dissected, post-fixed overnight with 4% formaldehyde at 4 °C and stored in PBS with 0.025% sodium azide (Sigma-Aldrich). Mice used for dissection of calvariae were perfused only with PBS and these samples were used for an osteoclastogenesis (TRAP) assay (see below). Samples used for hematoxylin and eosin (H&E) staining were decalcified in 250 mmol·L^−1^ EDTA in phosphate buffer (pH 7.4) at 4 °C. Decalcification was monitored by weekly X rays until no evidence of calcified tissue was observed. Tissue was then embedded in paraffin, cut into 5 µm sections and stained. Microscopic images were obtained with a Leica Aperio Scancope (Leica, Buffalo Grove, IL) or a Zeiss Axioplan 2 microscope equipped with an AxioCam HRc camera (Carl Zeiss, Inc., Thornwood, NY).

### Tartrate-resistant acid phosphatase activity assay (TRAP)

Calvariae were dissected, rinsed in ice-cold PBS, fixed in acetone citrate for 30 s, washed briefly in PBS and transferred to a pre-warmed garnet/tartrate solution from an Acid Phosphatase kit (Sigma-Aldrich). Calvariae were stained at 37 °C, protected from light, and assessed every 5 min. Stained specimens were stored in 70% ethanol and photographed using a dissection microscope.^30,35^

### Colony-forming efficiency assay and cell culture

Eight- to twelve-week-old, age and sex-matched mice were euthanized, and femora and tibiae were aseptically removed and cleaned of soft tissues. Marrow cavities of the bones were flushed into growth medium [α-minimum essential medium (α-MEM), 2 mmol·L^−1^ glutamine (Invitrogen, Carlsbad, CA), penicillin (100 U·mL^−1^), streptomycin sulfate (100 μg·mL^−1^; Invitrogen), 2-mercaptoethanol (0.1 mol·L^−1^), and 20% lot-selected non-heat-inactivated fetal bovine serum] using a nutrient medium-filled syringe attached to a 23 G needle. To determine CFE, a freshly prepared single-cell suspension of bone marrow cells was plated at low (clonal) density in 25-cm^2^ plastic culture flasks (4 × 10^4^ nucleated cells per cm^2^) with growth medium and cultured for 10 days at 37 °C, 5% CO_2_ without changing the medium. Next, flasks were rinsed once with Hanks’ Balanced Salt Solution (GIBCO-Life Technologies, Grand Island, NY), fixed with 100% methanol for 30 min, and stained with an aqueous solution of saturated methyl violet for 30 min. Flasks were rinsed three times with distilled water and dried overnight. Dried, stained colonies (>50 cells) were counted using a dissecting microscope, as described.^60^

To establish non-clonal BMSC cultures, single-cell suspensions of bone marrow (6–8 × 10^7^ nucleated cells) were also plated into 75-cm^2^ flasks in growth medium and cultured for 7 days at 37 °C, 5% CO_2_. Adherent cells were then detached by incubating for 30 min at 37 °C in collagenase type IV (2 mg·mL^−1^ in α-MEM, Invitrogen), and then for 5 min at 37 °C in trypsin (0.05% in 0.53 mmol·L^−1^ EDTA, Invitrogen). Cultures were maintained for up to 3 weeks (3 passages) with medium changes every 3 days.^35,61^ Cultures were depleted of CD45^+^ and CD11b^+^ cells using a magnetic sorting system following the manufacturer’s instructions (MACS, Miltenyi Biotec Inc., Auburn, CA, USA), reseeded, and used 24 h later.^62^

### Ectopic bone/marrow organ (ossicle) formation

2 × 10^6^ BMSCs/SSCs were suspended in 15 µL of standard growth medium and absorbed into 7 mm × 5 mm × 5 mm collagen sponge cubes (Gelfoam^®^, Pfizer, New York, NY). NSG immunodeficient mice (NOD-scid IL2Rgnull, Jackson Laboratory) were anesthetized with isoflurane (2%–5% in O2). After shaving the fur, a longitudinal incision of 1 cm was made on the back of the animals, and up to four transplants were placed subcutaneously into the flanks of the mice. The incision was then closed using surgical clips (Fig. 3b). Full body radiograms where taken every 2 weeks using an IVIS Lumina-XR in vivo imaging system to track transplant calcification. Eight weeks after surgery, mice were euthanized, and transplants were dissected and processed for image analyses. For μCT analyses, a volume of interest (VOI) corresponding to the trabecular content of the transplants was generated using a semi-automatic custom Scanco evaluation software script and analyzed using the script designed for trabecular bone. For the three-dimensional binarized reconstructions, samples were segmented using a relative threshold of 180/1 000, sigma 1/5 and support 2/9 using Scanco’s reconstruction software. After scanning, the ossicles were processed for histology as described above.

### Drilled hole bone defect

Eleven-month-old mice were anesthetized with isoflurane (2%–5% in O_2_). The surgical procedure used was a modification of a model previously described.^33^ Briefly, a 1 cm incision was placed on the lateral side of the thigh along the femur. The femoral diaphysis was exposed by pulling apart the muscle fibers with surgical forceps. Holes (0.8 mm) were created by using an electric surgical drill (Ideal Micro Drill, CellPoint Scientific, Gaithersburg, MD) and drilling through the periosteum and cortex at the lateral aspect of the mid-diaphysis. The holes were located immediately distal to the third femoral trochanter, which was used as an anatomical landmark (Fig. 4a). The region was continuously irrigated with sterile PBS to avoid thermal necrosis. Five mL of PBS was used to gently rinse bone fragments from the holes, with care taken not to dislodge the marrow. Muscles were realigned and incisions were closed with tissue glue (3 M Vetbond^®^, Saint Paul, MN). Animals were X-rayed (Lumina-XR, PerkinElmer) immediately after surgery to check the location of the drilled hole. 15 days after surgery, animals were euthanized, and tissue was processed as described below. The femora were scanned by μCT with a VOI selected as a cylindrical region of 800 µm in diameter with curved ends, to encompass the original bone defect and following the curvature of both the periosteum and endosteum of the femur. The newly formed bone in the defect was analyzed using a Scanco script designed for trabecular bone. Bones were subsequently processed for histological analysis.

### In vitro osteogenic differentiation and alizarin red staining

Osteogenic differentiation of BMSCs in vitro was performed in standard growth medium that was supplemented with 10^−8^ mol·L^−1^ dexamethasone, 1 mmol·L^−1^ β-glycerol phosphate, 100 µmol·L^−1^ l-ascorbic acid 2-phosphate, (D/P/A) (Sigma-Aldrich, St. Louis, MO, USA). Cultures were monitored daily by phase contrast microscopy for evidence of mineralization, and osteogenic medium was changed every 3 days. For alizarin red staining, cultures were fixed with cold 70% ethanol for 1 h at room temperature and stained with a 2% aqueous solution of alizarin red (Sigma-Aldrich). In some cases, the relative level of Ca^2+^ accumulation was quantified by elution of bound Alizarin Red with 0.5 ml of 5% SDS in 0.5 N HCl for 30 min at room temperature, and the absorbance was measured at 405 nm. For some experiments, DM-5 cells, a spontaneously immortalized murine BMSC cell line isolated in our laboratory^34,35^ were used. Cells were thawed and cultured as described above. In some experiments, DM-5 cells were cultured in medium in which dexamethasone was replaced with 100 ng·μL^−1^ of recombinant human BMP2 (B/P/A) (R&D Systems, Minneapolis MN).

### siRNA transfection

Immortalized DM-5 cells were used to knockdown *Sfrp2* expression with siRNA due to the ease in which they transfect and their proven osteogenic differentiation capacity in vitro and in vivo. Cells were plated in standard growth medium without penicillin and streptomycin such that they were between 30% to 50% confluent after 24 h. For 1 well of a 24 well dish, 20 pmols of either *Sfrp2*-siRNA (ID s73481, Invitrogen) or control (scrambled) siRNA (Silencer Select Negative Control #1, Invitrogen) were combined with 1.25 µL of Lipofectamine RNAiMAX (Invitrogen) in Opti-MEM^®^ in Reduced Serum Medium (Invitrogen), and added to the cells. For larger vessels, the amount of siRNA and Lipofectamine used was scaled up proportionally. The medium was not changed until at least 18 h after transfection.

### Treatment with recombinant murine Sfrp2 (rm-Sfrp2)

In some experiments*, WT* and *Sfrp2* KO BMSCs/SSCs depleted of C45^+^ and CD11b^+^ cells, or DM-5 cells silenced for *Sfrp2* expression, were treated with recombinant mouse Sfrp2 (rm-Sfrp2, 1 μg·mL^−1^, R&D Systems, Minneapolis, MN) or vehicle (phosphate buffered solution, PBS) added into fresh medium.

### RNA extraction and qRT-PCR

BMSCs/SSCs were harvested 3 days after the addition of osteogenic medium for determination of mRNA expression of *Runx2* and *Osx*, or after 24 h of treatment with rm-Sfrp2, for determination of mRNA expression of *Axin2*, *C-myc* and *Cyclin D1*. Immortalized DM-5 cells were harvested 3 days after the addition of osteogenic medium and 4 days after treatment with siRNA. RNA was isolated using RNeasy Mini Kits (Qiagen, Valencia, CA) and Qiashredder columns (Qiagen) according to the manufacturer’s protocol. When additional cleanup of the RNA was necessary, RNA Clean Up and Concentrator-5 columns (Zymo Research Labs, Irvine, CA) were used according to the manufacturer’s protocol. RNA was reverse transcribed by using SuperScript III First-Strand Synthesis System (Invitrogen). qRT-PCR analysis was carried out using iQ™ SYBR Green Supermix (Bio-Rad Laboratories, Hercules, CA) on a Bio-Rad CFX96™ Real-Time PCR Detection System (Bio-Rad Laboratories). cDNA quantification was carried out using the comparative cycle threshold method (∆∆CT) using *Gapdh* as a housekeeping gene. Primers used for amplification are listed in Supplementary Table 1.

### Western blot

Cultures were harvested 5 h after treatment with rm-Sfrp2 for protein extraction for determination of pLrp6 levels. Protein was extracted from cell cultures using PhosphoSafe Extraction Reagent (Novagen, Madison, WI) supplemented with 1 μg·mL^−1^ protease inhibitor cocktail (Roche). Protein extracts were centrifuged at 12 000 r·min^−1^ for 20 min at 4 °C, and equal amounts of lysate subjected to SDS-polyacrylamide gel electrophoresis using 4%–12% Bis-Tris precast polyacrylamide gels (NuPage, Invitrogen) and MOPS buffer (Invitrogen). Protein was then blotted onto polyvinylidene difluoride (PVDF) membranes (Immobilon-FL, Millipore, Darmstadt, Germany). Blots were blocked with Odyssey Blocking Buffer (Li-Cor, Lincoln, NE) and then incubated overnight with anti-pLrp6S1490 (1:1 000; Catalog #2568; Cell Signaling Technology, Danvers, MA). It is possible that the levels of pLrp6 (Ser1490) reported here also include pLrp5 (ser1493), as per the manufacturer’s description. The antibody used is expected to also detect this highly similar epitope. Hsp90 (1:1 000; sc-13119; Santa Cruz Biotechnology, Dallas, TX) labeling was carried out by incubating the blot at room temperature for 1 h. Protein bands were visualized by use of fluorescently labeled secondary antibody (1:10 000; Li-Cor), and scanned and quantified with an Odyssey scanner (Li-Cor). The relative protein expression levels were normalized to Hsp90 expression levels.

### Statistical analysis

The number of independent experiments and replicates are indicated in Supplementary Table 2. Data are expressed as mean ± SEM for all values. Results were evaluated using unpaired Student’s *t* tests, except for the drilled hole defect model, in which the *t* test was paired (comparing littermates). Analysis was performed using GraphPad Prism 6 software (GraphPad Software, La Jolla, CA). *P* < 0.05 was considered significantly different.

## Supplementary information

Supplementary material
