# Supplementary material for: Secreted frizzled related-protein 2 (Sfrp2) deficiency decreases adult skeletal stem cell function in mice
Source: Bone Res. 2021 Dec 2;9:49. doi: 10.1038/s41413-021-00169-7 (PMC8639730; doi:10.1038/s41413-021-00169-7)

***Secreted frizzled related-protein 2* (*Sfrp2*) deficiency decreases adult skeletal stem cell function in mice**

LF de Castro et al.

Supplementary Materials

Supplementary Table 1 - Primers used for qRT-PCR

Gene	Primer
Axin2	Forward 5′-CCGATTGCTGAGAGGAACTGGAAG-3′
	Reverse 5′-GCTGCTGCTGGGATCTGGAAGG-3′
C-myc	Forward 5′-TCCCACCCCGCCCCTGTC-3′
	Reverse 5′-CCACCGCCGCCGTCATCG-3′
Cyclin D1	Forward 5′-GCGTGGTGGCTGCGATGC-3′
	Reverse 5′-GAAAGAAAGTGCGTTGTGCGGTAG-3′
GAPDH	Forward 5′-TGCACCACCAACTGCTTAGC-3′
	Reverse 5′-GGCATGGACTGTGGTCATGAG-3′
Osterix	Forward 5-AGCACCAATGGACTCCTCTC-3
	Reverse 5-GGGTGGGTAGTCATTTGCAT-3
Runx2	Forward 5'-CGCCCCTCCCTGAACTCT-3'
	Reverse 5'-TGCCTGCCTGGGATCTGTA-3'
Sfrp1	Forward 5'-CTAGTGGCCTTGGATTGACC-3
	Reverse 5'-GTTGGCATGGTGAGTTTTCA-3

Sfrp2	Forward 5'-AAACCCTTTGTAAAAATGACTTCG-3'
	Reverse 5'-CAGCTTGTAAATGGTCTTGCTC-3'
Sfrp4	Forward 5'-ACCTGAGCAAAAACTACAGCTATG-3
	Reverse 5'-CTACCACAGTTGTGACCTCATTG-3

Supplementary Table 2 – Sex, age and number of animals or culture replicates used per experiment

	Assay	Figure	Sex	Age	Genotype	N
In vivo	**X-ray and µCT characterization**	1A, SF 1, SF 2A	Males	3 m	+/+	6
					-/-	8
				11 m	+/+	7
					-/-	6
			Females	3 m	+/+	5
					-/-	6
				11 m	+/+	5
					-/-	5
	**Calvarial TRAP staining**	SF 2B	Females	3 m	+/+	3
					-/-	3
	**Knee joint H&E**	1B,C	Males	3 m	+/+	5
					-/-	5
				11 m	+/+	3
					-/-	3
			Females	3 m	+/+	2
					-/-	2
				11 m	+/+	4
					-/-	5
	**Drilled defect regeneration**	3	Males	12 m	+/+	4
					-/-	4
			Females		+/+	2
					-/-	2
	**BMSC ectopic bone formation**	2B-E	Males	6 w	+/+	6
					-/-	5

	Assay	Figure	Replicates (N)	Experiment (N)
In vitro	**CFE, *Sfrp 1, 2* and *4* expression in in *Sfrp2* KO BMSCs**	2A, SF4	3	3
	**Osteogenesis in *Sfrp2* KO BMSCs and *Sfrp2-siRNA* DM-5 cells**	4		
	**Wnt pathway in *Sfrp2* KO BMSCs and *Sfrp2-siRNA* DM-5 cells**	5		

Supplementary Figure Legend

Supplementary Fig. 1. μCT analysis of femora of *WT* and *Sfrp2* KO littermates show no significant differences. μCT parameters of distal trabecular bone and mid-diaphyseal cortical bone measured in young (3 months) and old (11 months) males and females. Tb BV/TV (%) = Trabecular Bone Volume/Total Volume (%), Tb.Th = Trabecular Thickness, Tb.N = Trabecular Number, Tb.Sp = Trabecular Separation, C.Po = Cortical Porosity, C.Th = Cortical Thickness. Individual values of 5 or more are shown as white or black circles and blue bars represent the mean.

Supplementary Fig. 2. Skulls from *WT* and *Sfrp2* KO littermates show no significant anatomical differences, or differences in osteoclastic activity. ***A*** Representative μCT reconstructions of the skull of 4-month-old male mice showing no anatomical differences. ***B*** Representative photographs of TRAP staining of 3-month-old female calvariae show no increase in TRAP staining in the sutures between *WT* and *Sfrp2* KO mice.

Supplementary Fig. 3. Transfection of immortalized DM5 cells with *Sfrp2-siRNA* successfully decreases *Sfrp2* RNA levels for at least 7 days. qRT-PCR was used to assess the relative expression of *Sfrp2*. *Gapdh* was used as housekeeping gene for normalization. **$p<0.01$, ***$p<0.001$ vs. untreated control.

Supplementary Fig. 4. *Sfrp2*-deficient BMSCs/SSCs exhibit an increased expression of *Sfrp1* and *Sfrp4*. Expression levels of *Sfrp1*, *Sfrp2* and *Sfrp4* in BMSC/SSC cultures from *Sfrp2* KO and *WT* mice after 15 days of culture. *$p<0.05$, **$p<0.01$, ***$p<0.001$.

Supplementary Fig. 1

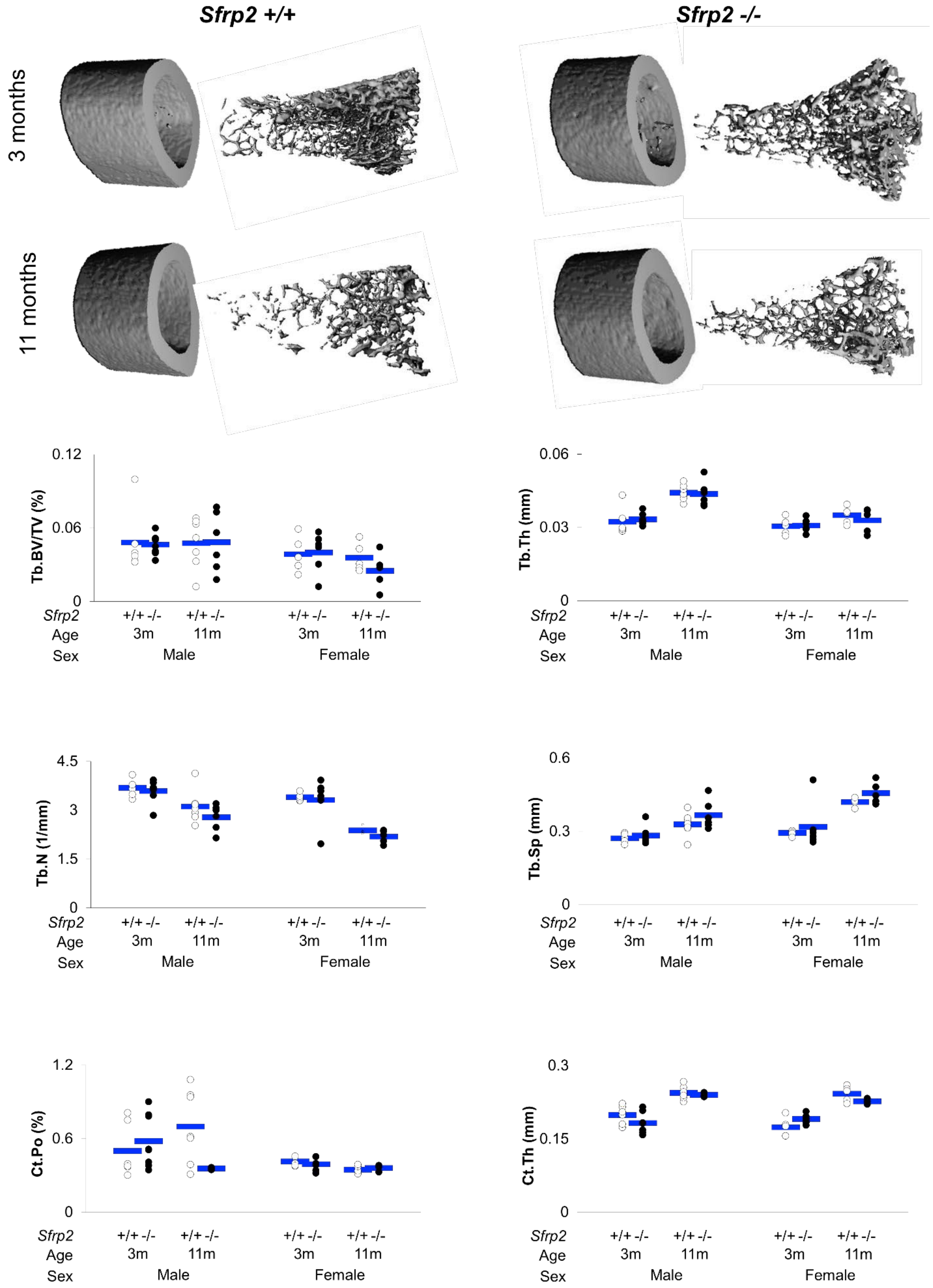

Supplementary Fig. 2

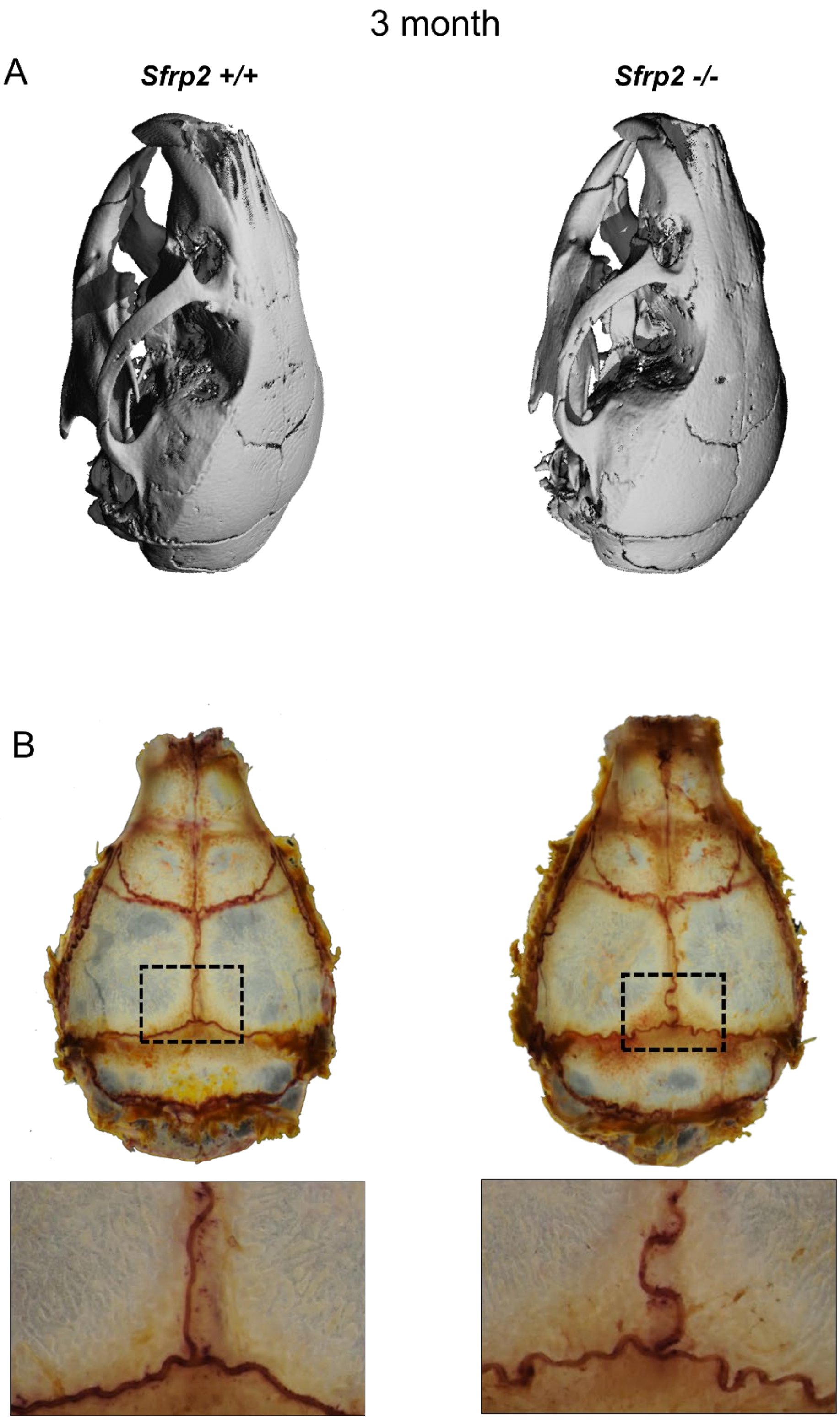

Supplementary Fig. 3

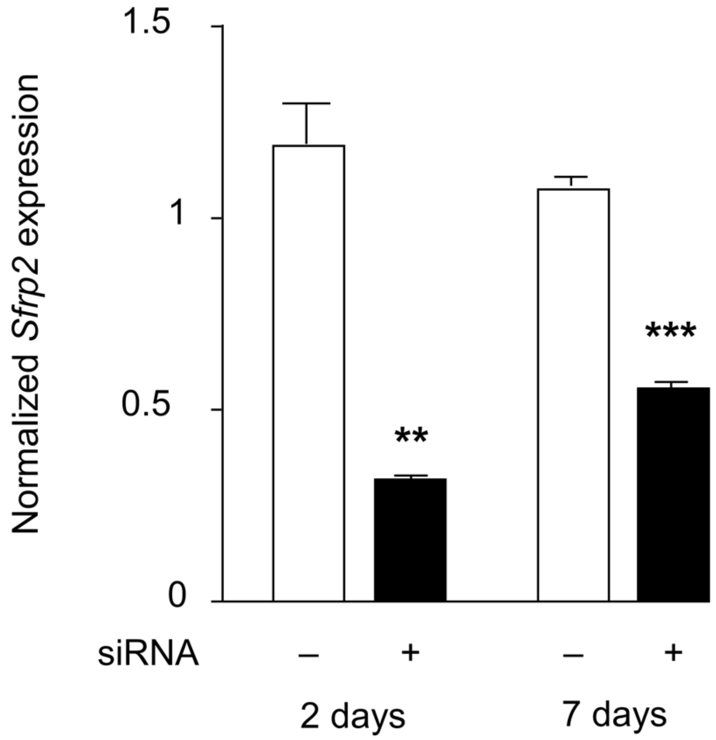

Supplementary Fig. 4

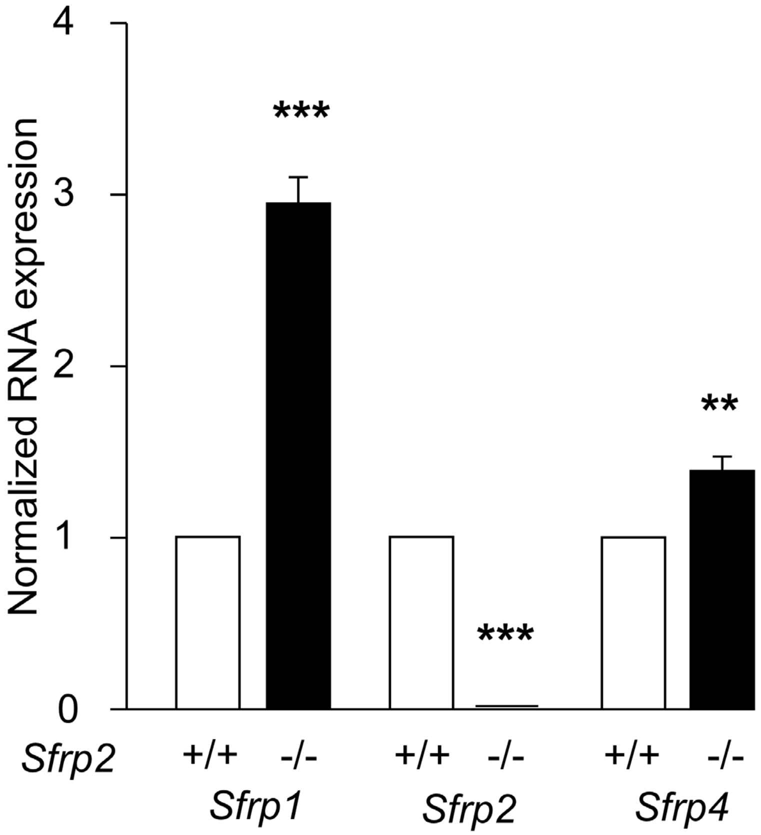

Supplement: Supplementary file 1 — Supplementary material [file 41413_2021_169_MOESM1_ESM.pdf]
